# From Pixels to Prediction: Developing Integrated AI Foundation Models for Personalized Thyroid Cancer Care

**DOI:** 10.3390/cancers18071155

**Published:** 2026-04-03

**Authors:** Jae Hyun Park, Younghyun Park, Yong Moon Lee, Sejung Yang, Jong Ho Yoon

**Affiliations:** 1 Department of Surgery, Yonsei University Wonju College of Medicine, Wonju 26492, Republic of Korea; jhoney@yonsei.ac.kr; 2 Department of Medical Informatics and Biostatistics, Yonsei University Wonju College of Medicine, Wonju 26492, Republic of Korea; pyh2908@yonsei.ac.kr; 3 Department of Pathology, College of Medicine, Dankook University, Cheonan 31116, Republic of Korea; 12200301@dankook.ac.kr

**Keywords:** thyroid cancer, personalized treatment, artificial intelligence (AI), foundation model

## Abstract

**Background**: Thyroid cancer incidence continues to rise globally, yet current diagnostic methods, reliant on ultrasound-guided fine-needle aspiration, suffer from substantial inter-observer variability and indeterminate results. **Objective**: This review explores the transformative potential of integrated artificial intelligence (AI) foundation models in thyroid cancer management. We propose a paradigm shift using foundation models—large-scale, multimodal architectures pre-trained on diverse datasets—to bridge the gap between initial pixels and long-term prognostic prediction. **Proposed Models**: We introduce two integrated conceptual frameworks: ThyroSight-Prognos for high-precision assessment in specialized tertiary settings and SonoPredict-AI for cost-effective screening in primary care. **Key Innovations**: By synthesizing data from ultrasound, pathology (WSI), genomics, and clinical parameters through explainable AI (XAI), these models aim to reduce unnecessary surgeries and personalize treatment pathways. **Challenges and Outlook**: This paper addresses critical implementation challenges, including data heterogeneity, hardware requirements, and regulatory trust, ultimately providing a strategic blueprint for future multi-center prospective clinical validation to revolutionize thyroid care through precision oncology.

## Simple Summary

This study proposes using integrated AI foundation models to improve thyroid cancer diagnosis and treatment. Unlike previous narrow computer models, these advanced systems bridge the gap between initial medical imaging and long-term prediction by integrating multimodal data into specialized frameworks: ThyroSight-Prognos for specialized hospitals and SonoPredict-AI for cost-effective primary care. By making these models transparent through visual and clinical explainability tools (XAI), the study seeks to enhance trust and usability in hospitals. The potential impact is significant: reducing unnecessary surgeries and personalizing treatments while addressing technical feasibility, including specific hardware requirements and iterative clinician feedback cycles. For researchers, this work provides a strategic blueprint for future multi-center clinical validation, encouraging the integration of data-driven precision oncology into the global healthcare system.

## 1. Introduction

Thyroid cancer presents a significant and growing global health challenge. With incidence rates having tripled over the past three decades and mortality rates remaining stubbornly high despite therapeutic advancements, it is currently ranked as the fifth most diagnosed cancer among women aged 15–49 worldwide [1,2,3]. Despite sophisticated detection technologies, particularly advancements in imaging, malignancy in thyroid nodules frequently presents as clinically ambiguous cases [4,5,6], placing a considerable burden on personalized risk stratification. The COVID-19 pandemic further exacerbated these challenges, creating a diagnostic backlog that revealed how persistently undertreated cases contributed to re-emerging challenges in subsequent years [7,8].

Traditional diagnostic paradigms, heavily reliant on ultrasound-guided fine-needle aspiration (FNA) and classic risk stratification systems like the American Thyroid Association (ATA) guidelines, demonstrate notable limitations in the era of precision medicine. Inter-observer variability in ultrasound interpretation is substantial, leading to a 20–40% discordance rate when applying systems like Thyroid Imaging Reporting and Data System (TI-RADS) [9,10]. Moreover, cytology results falling within indeterminate Bethesda III categories remain a diagnostic “gray zone” with a wide range of malignancy risk, making confident prediction challenging [6,10]. This inherent diagnostic uncertainty frequently contributes to unnecessary invasive procedures, with an estimated 52% of thyroidectomies potentially being unnecessary fine-needle aspirations according to some estimates [9,10]—potentially leading to overtreatment in 7–15% of cases, while simultaneously missing the opportunity to identify aggressive variants early on [2,11].

In response, the emergence of artificial intelligence (AI) technologies has ignited a paradigm shift. Deep learning architectures, particularly Convolutional Neural Networks (CNNs) optimized for imaging analysis, now offer impressive accuracy; models for cytopathology slide analysis demonstrate capabilities exceeding 97% accuracy, often outperforming even senior pathologists in diagnostic precision [10,11]. Similarly, contemporary AI systems using refined nodule characterization in ultrasound images, including ensemble models like the version of ThyNet described, have achieved high area under the curves (AUCs) (0.922 vs. 0.839) in malignancy prediction compared to human radiologists [9]. Beyond initial diagnosis, machine learning algorithms are fundamentally redefining prognostic capabilities. For instance, Random Forest models incorporating key clinical features and lymph node metastasis ratios have achieved an impressive 85% accuracy in predicting 5-year recurrence, surpassing traditional staging systems [9,12]. AI’s ability to integrate multimodal data—imaging, clinical parameters, pathology, biochemical markers, and genomics—provides a more nuanced risk stratification crucial for personalized treatment planning [13,14]. This computational revolution arrives at a critical juncture, coinciding with a projected 29.9% global increase in thyroid cancer incidence by 2040 [3,15]. AI-assisted diagnostics are estimated to yield substantial cost savings, potentially saving $45,000 annually per institution through optimized biopsy triage alone [9], while AI predicting preoperative imaging needs and enabling predictions like extrathyroidal extension could potentially reduce thyroidectomy completion rates by 33%, thus minimizing complications [9,11].

Yet, significant challenges persist in the path to widespread clinical adoption. Heterogeneous data across institutions, algorithmic biases (particularly concerning underrepresented populations), and the inherent “black box” nature of complex deep learning models remain hurdles for clinical trust and seamless integration [16,17]. Addressing the “black box” nature of complex AI models is critical for gaining clinical acceptance. Here, the integration of explainable AI (XAI) frameworks marks a pivotal advancement [9,17]. Techniques such as LIME (Local Interpretable Model-agnostic Explanations) and SHAP (SHapley Additive exPlanations) enhance prediction transparency, significantly contributing to building clinician understanding and trust by providing clinically interpretable prediction pathways. These developments pave the way for hybrid foundation models that intelligently combine Convolutional Neural Networks for image analysis with tree-based algorithms for risk prediction, creating systems capable of end-to-end diagnostic/prognostic solutions with enhanced clinical transparency [17]. As healthcare stands on the brink of this AI-driven transformation, a comprehensive understanding of the models’ capabilities, limitations, and implementation barriers becomes paramount for clinicians navigating this new era of computational oncology. The emergence of AI technologies has ignited a paradigm shift. The field is transitioning from task-specific “narrow AI” to foundation models. Unlike conventional machine learning used in radiomics, foundation models utilize transformer-based architectures and self-supervised learning on massive datasets, allowing for multi-task adaptability and cross-modal reasoning that were previously unattainable.

## 2. Redefining Thyroid Cancer Care with Smart Models

Our analysis begins by examining the current landscape of AI applications in thyroid cancer, highlighting several notable diagnostic and prognostic models. In the realm of diagnosis, systems primarily focus on interpreting imaging and cytopathology. The integration of artificial intelligence (AI) into the diagnostic workflow for thyroid cancer represents a significant step forward, promising improvements in malignancy detection, risk stratification, and overall clinical efficiency. Recent developments have leveraged diverse imaging modalities, including ultrasonography (US) and cytopathology, and have begun to incorporate explainable AI (XAI) frameworks to enhance both diagnostic accuracy and clinician trust.

To ensure conceptual clarity, it is essential to distinguish “Foundation Models” from conventional machine learning (ML) pipelines. Conventional ML, such as standard Convolutional Neural Networks (CNNs) for radiomics or pathology image analysis, is typically trained on task-specific, smaller datasets and often requires manual feature engineering. In contrast, foundation models are large-scale architectures—frequently based on transformer-based models—pre-trained on vast and diverse multimodal datasets (e.g., millions of medical images, text from electronic medical records, and genomic sequences). These models possess unique “zero-shot” or “few-shot” adaptability, meaning they can be adapted to various clinical tasks—such as rare variant detection or recurrence prediction—with minimal additional fine-tuning, representing a fundamental departure from the static, single-purpose algorithms of the past.

A comparative analysis of several key models illustrates the current state of the art and highlights ongoing challenges (Table 1).

The BETNET model [11] exemplifies a lightweight Convolutional Neural Network (CNN) specifically optimized for real-time ultrasound image analysis. This model has demonstrated promising performance, achieving an area under the curve (AUC) of 0.922 in differentiating benign from malignant nodules with a high specificity of 92.2%. Its compatibility with portable ultrasound devices and rapid processing time (<0.5 s per image) represent significant practical strengths. However, a limitation has been noted in its reduced accuracy for subcentimeter nodules, where the AUC drops to 0.79 [11].

Another significant diagnostic model is the Thyroid-Cytopathology-Specific CNN (TCS-CNN) with Attention-based Deep Multiple Instance Learning (AD-MIL) [18], which applies a patch-based CNN combined with Attention-based Multiple Instance Learning to whole-slide cytopathology images. This approach has shown remarkable accuracy, achieving 97% accuracy in Bethesda classification and notably reducing the rate of indeterminate diagnoses by 40%. A key innovation of this model is the use of Gradient-weighted Class Activation Mapping (Grad-CAM) visualizations to highlight malignant regions within cytopathology images, aiding pathologists by pointing to suspicious features like nuclear grooves or pseudoinclusions. Despite its capabilities, a challenge noted is the requirement for manual filtering of non-diagnostic patches, which could potentially increase pathologist workload [18].

Furthermore, preliminary work on a model also termed ThyNet has already demonstrated the potential of integrating ultrasound and cytology data using a deep CNN, showing impact in reducing FNAs by 30% while maintaining a 95% negative predictive value (NPV) [19]. Their multi-center validation study involving 4305 cases reported a superior AUC of 0.922 compared to the 0.839 achieved by radiologists [19].

While the preceding models primarily address diagnosis, the comparative analysis also highlights AI advancements relevant to patient prognosis. The XAI-LIME model focuses on predicting thyroid cancer recurrence risk. It combines Random Forest (RF) algorithms with Local Interpretable Model-agnostic Explanations (LIME), providing an explainable AI framework [20]. Using clinicopathological data, this model achieved a high accuracy of 96% in external validation and identified key predictors of recurrence, notably thyroglobulin (Tg) levels and the lymph node metastasis ratio (LNR) [20]. While the explainability of this model is a significant advantage for clinical adoption, its reliance on high-performance GPUs may present a limitation for deployment in resource-constrained or rural settings due to computational costs.

Collectively, these models underscore the substantial potential of AI to refine thyroid cancer diagnostics and prognostics. Nevertheless, they also expose persistent challenges that must be addressed for successful real-world implementation. These include ensuring generalizability across diverse patient populations and clinical sites, managing computational resource demands, and achieving seamless integration into heterogeneous clinical workflows. Bridging the gap between algorithmic innovation and practical application will necessitate dedicated future efforts focused on developing large, multi-institutional datasets and standardizing imaging protocols.

## 3. How Explainable AI Is Revolutionizing Thyroid Cancer Care

A critical consideration for the clinical translation and adoption of AI models, particularly in high-stakes applications such as thyroid cancer management, is their explainability. As complex “black-box” models become more prevalent, understanding *how* they arrive at a prediction is paramount for fostering trust among clinicians and enabling critical assessment [9,17]. In our proposed foundation models, XAI is not merely an optional feature but a core design objective—the “linchpin” of clinical adoption. Researchers developing the AI models discussed in this context have employed various techniques to enhance their transparency and interpretability.

One prominent approach utilized in the differentiated thyroid cancer (DTC) recurrence prediction model developed by Al-Sayed Ahmad and Haddad is Local Interpretable Model-agnostic Explanations (LIME) [20]. LIME provides *localized* explanations, focusing on approximating the behavior of the complex model around a *specific data point* to explain individual predictions. This allows clinicians to understand the key features that most significantly influenced a particular outcome for a given patient [20]. For example, LIME highlighted features like high “Thyroid Function” and low “Pathology” scores as supporting a prediction of non-recurrence, while high “M” (Metastasis) and “T” (Tumor Size) values contributed to recurrence predictions [20]. This ability to clarify individual case predictions is invaluable for clinical decision-making and building confidence in the model’s output [20].

Complementing the local insights provided by LIME, Al-Sayed Ahmad and Haddad also employed Morris Sensitivity Analysis for their DTC recurrence model [20]. This technique offers a *global* perspective, identifying which features have the most substantial *overall* impact on the model’s predictions across the entire dataset [20]. It calculates sensitivity indices (μ* for average impact and σ for variability/interaction) to rank feature importance [20]. In their study, “Response” and “Stage” were identified as primary global predictors, while features like “Pathology” and “Thyroid Function” were found to have minimal overall impact across the cohort, despite their potential influence in specific individual cases as highlighted by LIME. This dual approach with LIME and Morris Analysis provides a comprehensive understanding of both the model’s case-specific reasoning and its general behavior [20]. To complement the local insights provided by LIME and the global perspective offered by Morris Sensitivity Analysis, researchers can also employ SHAP (SHapley Additive exPlanations) for additional model transparency. For instance, SHAP values can clarify the relative contribution of key biochemical indicators—such as thyroglobulin (Tg) levels and lymph node metastasis ratio (LNR)—in 5-year recurrence prediction. This enables clinicians to directly validate the model’s logic against established clinical knowledge, further enhancing trust and interpretability.

For image-based diagnostic models, visual explainability techniques are particularly powerful. Gradient-weighted Class Activation Mapping (Grad-CAM) has been integrated into the TCS-CNN model developed by Oh et al. [18] to generate visual heatmaps overlaid onto cytopathology whole-slide images. These heatmaps effectively highlight the specific regions or pixels—such as irregular nuclear contours or microcalcifications—that the model focuses on during malignancy classification. Such visual explanations closely mimic the diagnostic reasoning of human pathologists, thereby bridging the gap between algorithmic output and clinical intuition [18].

Within the Attention-based Deep Multiple Instance Learning (AD-MIL) framework used by Oh et al. in their TCS-CNN, attention scores serve as another layer of explainability [18]. The attention mechanism inherently assigns weights or scores to individual instances (small patches) within a larger image (bag) [18]. These scores explicitly reflect the learned importance of each small patch in contributing to the overall bag-level diagnosis [18]. Visualizing these attention scores provides insight into the model’s aggregation process, showing which specific sub-regions within the whole slide were deemed most critical for the final prediction [18].

Beyond individual clinician trust, transparency and interpretability are increasingly becoming mandatory for regulatory alignment. When both visual attention maps (via Grad-CAM) and feature-level importance scores (via SHAP or attention mechanisms) are provided simultaneously, AI outputs can be seamlessly integrated into standardized hospital decision-making workflows. Finally, we envision an iterative optimization cycle in which explainable outputs are continuously refined through Reinforcement Learning from Human Feedback (RLHF). By presenting these interpretable predictions to clinicians, a “clinician-in-the-loop” paradigm is enabled, where expert feedback is used to fine-tune the model’s weighting of clinical parameters, ensuring that the AI’s logic remains robust and clinically relevant over time.

Collectively, these diverse techniques—providing local insights (LIME), global perspectives (SHAP and Morris Analysis), and visual cues (Grad-CAM and attention scores)—work to demystify the “black-box” nature inherent in many complex AI models, fostering the responsible and safe adoption of AI in critical healthcare settings [9,18,20].

## 4. Predicting Thyroid Cancer’s Future

Moving beyond the initial diagnosis, accurately predicting the future trajectory of thyroid cancer—including the risks of metastasis, recurrence, and overall impact on survival—is fundamental to developing personalized patient management plans and optimizing treatment strategies. Machine learning (ML) models are increasingly demonstrating a superior capacity to provide more nuanced prognostic stratification compared to traditional staging systems by integrating diverse clinical, pathological, and imaging data [16,21].

A significant area of focus within AI research has been the prediction of metastatic spread, a critical factor influencing both treatment intensity and patient outcomes. Models have been specifically developed to anticipate various forms of metastasis.

**Lymph Node Metastasis (LNM)**, particularly in the cervical region, is a key area where AI algorithms have shown considerable success in prediction prior to surgery. For Central Lymph Node Metastasis (CLNM), models such as Gradient Boosting Decision Tree (GBDT) and eXtreme Gradient Boosting (XGBoost) have been employed, leveraging features derived from ultrasound and clinical data [21]. Xia et al., for instance, utilized a Probabilistic Neural Network (PNN) which achieved 88.4% accuracy in predicting CLNM [22]. Further enhancing predictive power, Chang et al. developed an integrated nomogram that combined deep learning (DL), clinical characteristics, and ultrasound features, achieving AUCs up to 0.829 upon external validation [22]. Regarding Lateral Lymph Node Metastasis (LLNM), models like Support Vector Machine (SVM) and K-Nearest Neighbors (KNN) have proven effective [21]. Xia et al. reported a particularly high accuracy of 94.7% for an SVM model in predicting LLNM [22]. Research by Feng et al. has further compared various ML models, highlighting the utility of different algorithms for LLNM prediction [21]. For Delphian Lymph Node Metastasis (DLNM), Random Forest (RF) models have been successfully applied [21]. Beyond ultrasound, deep learning models analyzing Computed Tomography (CT) images have also shown excellent performance in predicting LNM, with reported AUCs ranging from 0.8 to 0.84 [22].

**Distant metastasis** prediction is another crucial application, with AI being applied to forecast spread to remote sites like the lungs and bones, often utilizing large databases such as SEER. Liu et al. developed an RF model specifically for predicting lung metastasis, reporting a remarkably high AUC of 0.991 [21,22]. In another study, also by Liu et al., an RF model trained on SEER data was used to predict bone metastasis, achieving an AUC of 0.917 and a specificity of 90.5% [21,22]. Across various metastasis prediction models, key identified predictors frequently include patient age, primary tumor characteristics (such as TNM stage, size, location, and grade), and specific lymph node features like size and the presence of microcalcifications [21,22].

Beyond metastasis, AI models are also crucial for predicting long-term outcomes like disease **recurrence and survival**. Predicting recurrence after initial therapy is a cornerstone of long-term management, and ML models aim to identify patients at elevated risk [23,24]. Park et al. compared five different ML models using clinico-pathologic factors for predicting recurrence in Papillary Thyroid Carcinoma (PTC) and found that Decision Tree (DT), LightGBM, and stacking models achieved accuracies exceeding 90% [12]. Kil et al. specifically explored the use of deep learning applied directly to *preoperative ultrasound images* to predict tumor recurrence [25]. Furthermore, Kim et al. employed Inductive Logic Programming (ILP) to integrate pathological and genetic information effectively for recurrence prediction in well-differentiated thyroid cancer [26].

In the domain of **survival prediction**, AI models are being developed to forecast patient outcomes over longer periods. Jajroudi et al. compared Artificial Neural Network (ANN) and logistic regression models for predicting 1-year survival in thyroid cancer patients, finding the ANN model to be superior [22]. Mourad et al. used Multi-Layer Perceptron (MLP) models trained on SEER data to predict 10-year survival rates [22]. Other algorithms, including XGBoost and Partitioning Around Medoids (PAM), have also been successfully applied for prognostic prediction, particularly in specific thyroid cancer subtypes like Follicular Thyroid Carcinoma (FTC) and other well-differentiated cancers [21,22]. Predictors frequently highlighted in both recurrence and survival models encompass sociodemographic factors (e.g., marital status), details of the initial treatment (e.g., type of surgery), tumor characteristics (including size and lymph node ratio—LNR), and postoperative biochemical markers (such as thyroglobulin levels, anti-thyroglobulin antibodies, BMI, and TSH levels [21,22].

These AI-driven prognostic models collectively demonstrate significant potential to refine follow-up protocols, guide the selection of adjuvant therapies, and provide patients with more accurate information regarding their long-term outlook. This capability represents a key advancement towards personalized care in thyroid oncology. However, the challenges previously discussed, including data heterogeneity, ensuring model generalizability across diverse populations and institutions, and the critical need for rigorous prospective validation, continue to be important considerations.

## 5. How AI Uses Patient Profiles to Predict Thyroid Cancer Outcomes

Effective prognostication in thyroid cancer relies heavily on the integration of comprehensive patient information. The AI models designed for predicting patient outcomes, such as metastasis, recurrence, and survival, leverage a wide array of data types drawn from diverse clinical and pathological sources [21,27,28,29,30]. The specific combination of data variables employed can vary depending on the model’s particular objective and the design of the study, but several key categories of information are frequently integrated to build comprehensive patient profiles.

Firstly, fundamental **patient demographics and characteristics** play a crucial role. This includes basic information such as age, sex, and race [21,25]. More specific lifestyle factors like smoking status, alcohol drinking status, and marital status have also been incorporated into prognostic models [21,25].

Secondly, the presence of **comorbidities** is often considered as it can influence treatment decisions and outcomes. Frequently included comorbidities are diabetes, hypertension, and Hashimoto’s thyroiditis [21,25].

Detailed **tumor-related variables** concerning the primary tumor are essential for risk stratification. These variables include the histological type of the cancer (e.g., Papillary Thyroid Carcinoma (PTC), Follicular Thyroid Carcinoma (FTC), Medullary Thyroid Carcinoma (MTC), Anaplastic Thyroid Carcinoma (ATC), Follicular Variant of Papillary Thyroid Carcinoma (FV-PTC)), as well as tumor diameter or size, and the presence of multiple tumor foci (multifocality) [12,21,25]. The location of the tumor within the thyroid and the extent of extrathyroidal extension (ETE) are also critical factors [21,25]. Furthermore, components of the TNM staging system, specifically the T stage, are consistently used [21,25]. Genetic information, such as BRAF V600E mutation status or general genetic profiles, is increasingly being included, though perhaps less commonly than other clinical variables in the reviewed prognostic models [21,25].

Information pertaining to **lymph nodes (LNs)** is equally vital for prognosis, particularly in predicting metastatic spread and recurrence. This includes details on the type of LN dissection performed (none, central, or lateral), the number of lymph nodes dissected, the presence of extranodal extension (ENE), the lymph node metastasis ratio (LNR), and the N stage component of the TNM system. Specific ultrasound features of the lymph nodes themselves, such as size and the presence of microcalcifications, also contribute valuable data [21,25].

Beyond clinical and pathological reports, **imaging data** provides crucial visual information. This encompasses features extracted from preoperative ultrasound (US) images [12], including detailed characteristics like calcification patterns, nodule shape and margins, blood flow, location, size, and the ACR TI-RADS categorization [25]. Textual reports generated from ultrasound examinations are also utilized [21]. Furthermore, specific image features derived from Computed Tomography (CT) scans are incorporated, particularly for predicting lymph node metastasis [21].

**Biochemical and laboratory markers**, measured postoperatively, offer insights into residual disease and long-term risk. Key markers include non-stimulated thyroglobulin (Tg) levels and anti-thyroglobulin antibodies (TgAb) [12,21,25]. Thyroid Stimulating Hormone (TSH) levels are also relevant. Broader metabolic markers such as Body Mass Index (BMI) and lipid profiles (including triglyceride, cholesterol, and LDL and HDL levels) are sometimes included. Inflammatory markers like Neutrophil-to-Lymphocyte Ratio (NLR), Platelet-to-Lymphocyte Ratio (PLR), and Lymphocyte-to-Monocyte Ratio (LMR), as well as the Prognostic Nutritional Index (PNI), have been explored. Other general blood test results (e.g., hematocrit, ALT, RBC count) may also be considered [21,25].

Details regarding the **treatment and follow-up** provided are important for predicting outcomes. This includes the type of surgery performed (e.g., total thyroidectomy vs. hemithyroidectomy), specific details of any radioiodine therapy administered (frequency and dose), and follow-up data such as the patient’s vital status or the number of survived months [12,21,25].

Finally, **clinical outcome data** itself is fundamental for training and validating these prognostic models. This primarily consists of the patient’s recurrence status and the site of any recurrence, which serve as the target variables for prediction [12].

In summary, AI prognostic models for thyroid cancer harness a diverse pool of information from various sources including electronic medical records (EMRs), large clinical databases like SEER, blood tests, imaging reports, and pathology reports. By integrating these multimodal data types, these models construct comprehensive patient profiles, enabling the identification of complex patterns for more accurate and personalized prognostic predictions [21,25].

## 6. Integrating Diagnosis and Prognosis in Thyroid Cancer Care

Building upon the preceding discussions regarding advanced AI models for both the diagnosis and prognosis of thyroid cancer, we naturally arrive at the potential for developing a unified “foundation model.” Such a model would possess the capability to perform both diagnostic assessment and prognostic prediction, either concurrently or sequentially, within a single integrated framework. We propose that the conceptualization and development of such a system could fundamentally transform clinical workflows, moving towards a more holistic assessment from the initial evaluation. This would allow for not only determining the likelihood of malignancy but also forecasting the likely future behavior and trajectory of the disease for each patient.

The rationale underpinning the development of an integrated diagnostic–prognostic model is multi-faceted, encompassing both clinical and technical advantages. From a clinical perspective, a unified system offers the potential to significantly streamline the patient pathway. By providing comprehensive risk stratification earlier in the management process, it could facilitate more timely and personalized treatment planning, reduce diagnostic delays, and optimize resource allocation within healthcare systems. Technically, there is a compelling likelihood of synergistic information transfer between diagnostic and prognostic tasks. For instance, subtle morphological patterns identified by diagnostic models analyzing ultrasound images via CNNs like BETNET [11] or intricate cytopathological details detected by architectures such as TCS-CNN [18] may inherently hold prognostic significance that extends beyond what is captured by traditional clinical variables alone. Conversely, baseline clinical risk factors commonly utilized in prognostic models might subtly influence the model’s certainty or interpretation of specific diagnostic findings. An integrated framework is uniquely positioned to explicitly learn and leverage these complex interdependencies.

Several conceptual frameworks could serve as the architectural basis for such an integrated foundation model:

1. ***Sequential Pipeline*:** This represents a relatively straightforward approach involving a two-stage process. Initially, a diagnostic model, perhaps leveraging algorithms like BETNET for ultrasound images [11] or TCS-CNN for cytopathology [18], classifies the nodule or lesion. The output of this diagnostic stage—which could be a malignancy probability score, a predicted Bethesda category, or specific extracted image features—is then fed as an additional input into a separate prognostic model. This prognostic component, potentially employing ensemble methods such as Random Forest or XGBoost similar to those described in studies by Wang et al. or Liu et al. [21], would integrate these image-derived features alongside standard clinical, pathological, and biochemical data to generate a prognostic prediction. The modular nature of this design facilitates easier implementation and validation but might not fully capture the intricate, end-to-end interactions between diagnostic features and prognostic outcomes.
2. ***Multi-Task Learning (MTL) Framework*:** A more sophisticated approach involves developing a single, potentially complex neural network designed to simultaneously predict multiple outputs. Such an MTL model would be capable of accepting all relevant input data types, including imaging data, clinical variables, and biomarker levels. It would incorporate shared network layers to learn common, generalized feature representations from this multimodal input. These shared layers would then feed into separate, task-specific output branches designed to produce both a diagnostic classification and various prognostic predictions (e.g., recurrence risk, metastasis probability). This architecture has the potential to learn richer, shared feature representations and exploit correlations between the diagnostic and prognostic tasks more effectively than sequential approaches.
3. ***Ensemble or Fusion Models*:** This framework involves combining the outputs or features derived from distinct, potentially independently optimized, diagnostic and prognostic models. Various ensemble techniques or data fusion strategies could be employed to synthesize these outputs into a final, integrated assessment. This approach allows for leveraging the specific strengths of different model types—for instance, CNNs optimized for image analysis [11,21,31,32] and tree-based models well-suited for tabular data [21]. However, it requires careful calibration and weighting of the individual model contributions to achieve optimal performance.

While the technical feasibility of developing such an integrated foundation model is within reach using existing machine learning methodologies, its successful development and deployment face significant challenges. The foremost hurdle lies in data availability and quality. Robust foundation models necessitate comprehensive, large-scale datasets that meticulously link high-resolution diagnostic inputs (such as raw imaging files and detailed pathology reports) with accurate, long-term clinical follow-up data detailing outcomes like recurrence, metastasis, and survival for the *same* patients. Curating such rich, multimodal, and longitudinal datasets across multiple institutions is a formidable task, further complicated by inherent data heterogeneity across different sites and critical patient privacy concerns [16,21].

Beyond data, computational requirements would likely increase, particularly for architectures involving Multi-Task Learning or complex ensemble models that incorporate deep learning components for image analysis [11,18]. Maintaining model interpretability across an integrated system is another critical but challenging aspect; understanding precisely how specific diagnostic features contribute to the final prognostic score requires the application of advanced explainable AI (XAI) techniques systematically throughout the entire model pipeline [16]. Finally, the most rigorous standard for clinical adoption will be multi-center prospective validation. Demonstrating the clinical utility and safety of a system performing both diagnosis and prognosis simultaneously represents a higher bar for validation compared to assessing individual diagnostic or prognostic components in isolation.

Despite these significant challenges, the potential for an integrated foundation model offers a compelling vision for the future of AI in thyroid cancer care. By providing clinicians with a comprehensive diagnostic and prognostic assessment from the initial point of evaluation, such systems could yield more actionable insights. This capability has the potential to lead to truly personalized risk stratification and ultimately optimize treatment decisions for patients diagnosed with thyroid cancer.

## 7. Comprehensive and Cost-Effective Models for Thyroid Cancer Management

We are now prepared to propose integrated foundation model frameworks for thyroid cancer management. These frameworks are conceptualized with real-world clinical application in mind, carefully balancing state-of-the-art performance with critical considerations of cost-effectiveness. We propose two distinct yet potentially complementary models (Table 2).

We clarify that ThyroSight-Prognos and SonoPredict-AI are currently proposed as conceptual frameworks and architectural blueprints. While grounded in current state-of-the-art performances, they serve as a strategic guide for future implementation.

### 7.1. Proposed Foundation Model 1: ThyroSight-Prognos (High-Accuracy, Comprehensive Assessment)

This model is designed for maximum diagnostic precision and detailed prognostic stratification, envisioned for deployment in tertiary care centers, specialized pathology laboratories equipped with advanced digital slide scanners, or research settings where the most comprehensive assessment is paramount for guiding complex treatment decisions.

#### 7.1.1. Components

1. **Diagnostic Core:** At its core, ThyroSight-Prognos utilizes the TCS-CNN model with Attention-based Multiple Instance Learning (AD-MIL) [18]. This leverages the high accuracy (97%) and explainability provided by techniques like Grad-CAM and Attention scores for analyzing high-resolution whole-slide images (WSIs) derived from fine-needle aspiration cytology (FNAC) or histology samples [18].
2. **Prognostic Engine:** Integrated with the diagnostic output is a Random Forest (RF) model serving as the prognostic engine. This RF model is trained on a comprehensive set of clinicopathological variables, biochemical markers (such as non-stimulated Tg and LNR), and potentially incorporating genomic data. Drawing inspiration from models that have achieved high AUCs for predicting recurrence (e.g., AUC 0.85) and metastasis [21,22], this component provides detailed risk assessment.

#### 7.1.2. Rationale

The primary rationale for ThyroSight-Prognos is to offer the most precise diagnostic classification coupled with the most comprehensive prognostic assessment possible. This enables clinicians to obtain detailed recurrence and metastasis risk reports based on integrated high-fidelity pathological analysis and extensive clinical data, which is crucial for guiding individualized follow-up intensity and complex treatment choices.

#### 7.1.3. Cost-Effectiveness and Optimization

1. **Hardware:** This model requires a significant initial investment in high-resolution WSI scanners for digitizing slides and powerful GPU workstations to process the large and complex image data using TCS-CNN. However, the RF component for prognostication runs efficiently on standard CPUs.
2. **Optimization Strategy:** Cost-effectiveness for this high-performance model focuses on justifying the initial investment through downstream savings. Strategies include implementing highly efficient TCS-CNN code and exploring cloud-based processing options on secure, HIPAA-compliant platforms to mitigate upfront hardware costs. Federated learning frameworks are essential for multi-institutional model training using aggregated, de-identified features derived from patient data without sharing raw images or sensitive information, thereby enhancing generalizability while rigorously respecting privacy. The high cost is justified by the potential to significantly reduce unnecessary or inappropriate thyroidectomies, optimize adjuvant therapy use, and avoid complications by providing highly accurate diagnosis and prognosis.

#### 7.1.4. Clinical Workflow

**Figure 1:**
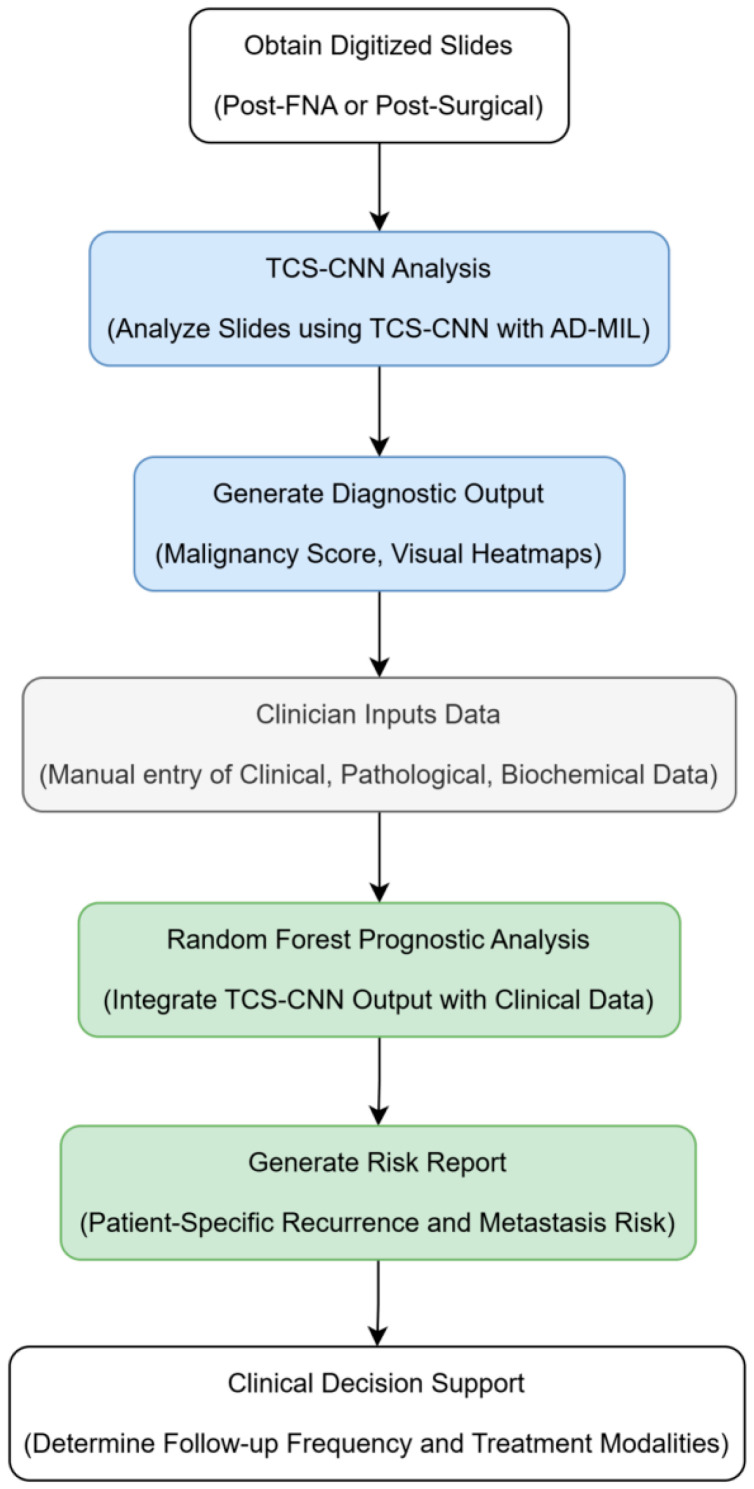
Clinical workflow for proposed foundation model 1: ThyroSight-Prognos (high-accuracy, comprehensive assessment).

In practice, post-FNA or post-surgical digitized slides would be analyzed by the TCS-CNN component, providing a malignancy score and visual heatmaps highlighting suspicious areas. This pathological output, combined with comprehensive clinical, pathological, and biochemical data manually entered by the clinician into the Electronic Medical Record (EMR) or a dedicated interface, would then be fed into the RF prognostic engine. This engine generates a detailed, patient-specific recurrence and metastasis risk report, directly supporting the clinician in determining optimal follow-up frequency and treatment modalities (Figure 1).

### 7.2. Proposed Foundation Model 2: SonoPredict-AI (Cost-Effective Screening and Initial Prognosis)

In contrast, this model is designed for broader accessibility and cost-effectiveness, suitable for primary care physicians, endocrinology clinics, and settings with limited resources. It prioritizes efficient initial risk stratification directly at the point of care, aiming to optimize resource use and reduce unnecessary procedures early in the patient pathway.

#### 7.2.1. Components

1. **Diagnostic Core:** SonoPredict-AI utilizes BETNET (11) or a similar lightweight, efficient CNN architecture specifically optimized for ultrasound (US) image analysis. Its focus is on providing real-time or near-real-time US nodule classification and malignancy risk assessment (e.g., achieving an AUC of 0.922 for benign vs. malignant differentiation) [22].
2. **Prognostic Engine:** This model employs a Random Forest (RF) model for initial prognostication. It relies primarily on readily available preoperative clinical data and key US features, potentially supplemented with basic postoperative information such as initial TNM stage and initial postoperative thyroglobulin (Tg) levels to provide a preliminary risk estimate.

#### 7.2.2. Rationale

The core rationale for SonoPredict-AI is its potential for widespread adoption and its ability to facilitate efficient, low-cost initial risk stratification directly from ultrasound evaluation. By accurately identifying low-risk or benign nodules early on, it aims to significantly reduce the number of unnecessary FNAs and streamline patient referrals, thereby alleviating downstream costs and patient anxiety [18].

#### 7.2.3. Cost-Effectiveness and Optimization

1. **Hardware:** BETNET’s lightweight architecture allows deployment on standard clinic PCs or potentially direct integration into modern US machines or existing PACS/EMR systems. The RF component requires minimal computational resources. Consequently, the overall hardware costs for this model are substantially lower than for ThyroSight-Prognos.
2. **Optimization Strategy:** Optimization focuses on seamless integration with existing clinical equipment and EMR systems to minimize workflow disruption. Pre-trained, validated models could be deployed locally or accessed securely via cloud services. The primary cost savings are derived from the significant reduction in the rate of FNAs for benign or very low-risk nodules (potentially a 30–40% reduction according to some estimates [18,22]) identified accurately by the US AI, which in turn reduces downstream expenses and improves patient experience.

#### 7.2.4. Clinical Workflow

**Figure 2:**
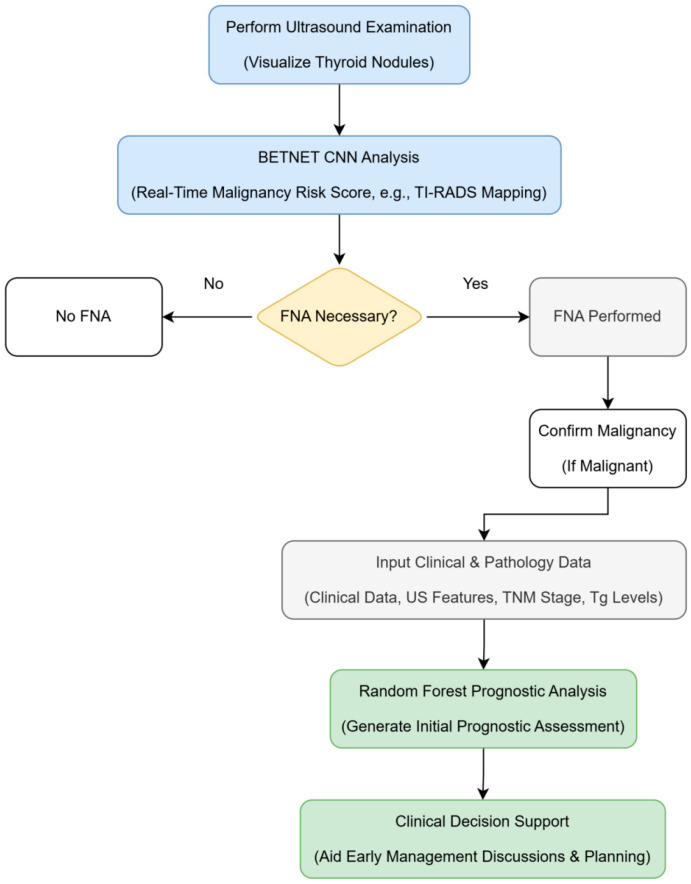
Clinical workflow for proposed foundation model 2: SonoPredict-AI (cost-effective screening and initial prognosis).

The workflow for SonoPredict-AI is centered during the US examination. The AI provides a real-time or immediate malignancy risk score for visualized nodules, potentially mapping directly to categories like TI-RADS. This information assists the radiologist or clinician in making informed decisions regarding the necessity of FNA. If malignancy is confirmed later (e.g., via FNA), readily available clinical, US, and initial pathology data can be input into the linked RF module to generate an initial prognostic assessment, aiding early management discussions and planning (Figure 2).

These two proposed foundation models, ThyroSight-Prognos and SonoPredict-AI, represent different yet valuable approaches positioned on the performance–cost spectrum. SonoPredict-AI serves as an effective, cost-optimized initial filter, streamlining assessment and resource utilization at the initial point of patient contact. In parallel, ThyroSight-Prognos provides a high-fidelity, comprehensive assessment crucial for navigating complex cases or providing definitive postoperative risk stratification. In an ideal, integrated clinical system, the output from SonoPredict-AI at the initial evaluation could potentially inform the urgency and necessity of proceeding to FNA and subsequent, more detailed analysis potentially provided by ThyroSight-Prognos. This tiered approach creates a synergistic pathway for optimizing the diagnostic and prognostic journey in thyroid cancer care, leveraging AI capabilities strategically across different levels of healthcare provision. However, to rigorously evaluate the quantitative clinical benefits of the proposed models—including reductions in cost, unnecessary surgeries, and patient burden—multi-center prospective validation studies are essential.

### 7.3. Practical Considerations and Computational Budget

To address the technical feasibility and quantitative rigor required for clinical translation, we provide “order-of-magnitude” estimates for the computational infrastructure needed to support these frameworks (Table 3). These estimates are derived from benchmark performances of representative foundation model architectures in oncology and are intended to guide institutional budgeting and deployment planning.

The primary rationale for ThyroSight-Prognos is to offer the most precise diagnostic classification coupled with a high-fidelity prognostic engine. This requires significant initial investment in high-resolution WSI scanners and powerful GPU workstations to process large-scale multimodal data. However, the high cost is justified by the potential to significantly reduce unnecessary total thyroidectomies and optimize adjuvant therapy use.

Conversely, the core rationale for SonoPredict-AI is its potential for widespread adoption through its lightweight architecture. By allowing deployment on standard clinic PCs or through secure cloud services, it aims to minimize workflow disruption. The primary cost savings are derived from the significant reduction in the rate of FNAs for benign or very low-risk nodules, which in turn reduces downstream expenses and improves the overall patient experience. The workflow for SonoPredict-AI is centered during the US examination, providing real-time malignancy risk scores that assist the radiologist in making informed decisions regarding the necessity of FNA without the need for expensive on-site server infrastructure.

## 8. Discussion

By synergizing the insights gained from advanced diagnostic tools, such as TCS-CNN or BETNET, with the powerful predictive capacities of integrated algorithms like Random Forest, these proposed models hold the promise of significantly impacting patient care, the practices of clinicians, and the broader healthcare landscape.

The most compelling advantages of these foundation models lie in their direct impact on patient care. Firstly, they promise enhanced diagnostic accuracy and a reduced patient burden. By providing more precise initial stratification, particularly in differentiating benign and malignant nodules using multimodal data, these models can lead to a significant reduction in unnecessary fine-needle aspirations (FNAs) and diagnostic surgeries [10,11]. This, in turn, directly minimizes patient anxiety and discomfort while alleviating the healthcare costs associated with avoidable invasive procedures [18]. Secondly, for clinicians, these foundation models function as powerful enhanced decision support systems, providing data-driven insights that can bolster diagnostic confidence and refine treatment planning. By automating aspects of complex image analysis and risk calculation, these models can streamline clinical workflows and alleviate the burden of repetitive tasks, freeing up valuable clinician time for more complex decision-making and direct patient interaction [11,18].

Despite these promising advantages, current AI systems for thyroid cancer still exhibit three fundamental gaps that limit their real-world impact: (1) task fragmentation, with most models focusing solely on diagnostic classification without linking to long-term prognostic endpoints; (2) data isolation, failing to exploit the synergy among ultrasound, pathology, genomics, and clinical records; and (3) deployment inflexibility, as they are rarely optimized for the differing hardware and workflow constraints of tertiary versus primary care settings.

Our proposed integrated foundation models directly address these limitations. Built on large-scale pre-training and task-agnostic adaptability (zero-shot/few-shot learning), they represent a new frontier in medical AI. While many existing models in radiology and computational pathology remain limited to single-modality tasks or isolated report generation, our frameworks explicitly bridge initial imaging with long-term prognostic trajectories. Specifically, ThyroSight-Prognos offers a high-fidelity multimodal solution for specialized tertiary care, while SonoPredict-AI provides a lightweight, cost-effective screening tool for primary care. This hierarchical and comprehensive approach is designed to meet the diverse needs across the entire thyroid cancer care pathway, going beyond the narrow scope of existing work.

Despite these promises, several technical and implementation barriers must be addressed. Reviewers of this work have rightly pointed out challenges such as data heterogeneity and imaging protocol variability across different institutions [16,17]. Future efforts must prioritize robust data harmonization techniques and the implementation of federated learning frameworks to allow models to learn from diverse global cohorts without compromising patient privacy. Furthermore, the challenge of “missing modalities”—for instance, cases where genomic data or certain laboratory markers are unavailable—must be addressed through architectural designs that can maintain performance with incomplete data streams.

To ensure the long-term reliability and clinical relevance of our proposed frame-works, we propose an iterative optimization cycle centered on Reinforcement Learning from Human Feedback (RLHF). Ouyang et al. [33] showed that RLHF can systematically align large language models with human preferences, improving instruction following and reducing harmful behaviors, which provides a conceptual template for medical LLMs embedded in thyroid cancer workflows. Complementarily, Wong et al. [34] introduced a Bayesian optimization framework that scales RLHF using crowd-sourced feedback in code generation, underscoring the importance of feedback quality control and efficient sampling of informative cases. Translating these ideas into our setting, we envision structured, clinician-in-the-loop feedback (e.g., rating, correcting, or revising AI-generated reports and risk stratifications) that is periodically aggregated and used as a reward signal to refine the model parameters within a Learning Health System cycle, thereby keeping ThyroSight-Prognos and SonoPredict-AI aligned with real-world thyroid cancer practice.

Finally, we acknowledge the current gap between conceptual proposal and clinical readiness. To address this, our future roadmap involves a step-wise validation plan starting from large-scale retrospective multi-center cohorts to real-time clinical utility studies. We emphasize that to rigorously evaluate the quantitative clinical benefits of these proposed models—including reductions in cost, unnecessary surgeries, and patient burden—multi-center prospective validation studies are essential. Only through such rigorous assessment can we transition these integrated AI foundation-style models from pixels to prediction, ultimately realizing the potential of data-driven precision oncology in thyroid cancer care.

## 9. Conclusions

The integration of AI foundation models points toward a promising future in thyroid oncology. By unifying tools like TCS-CNN and BETNET into a tiered system (ThyroSight and SonoPredict), we can streamline the journey from pixel-based diagnosis to long-term prediction. While technical challenges such as computational demand and data privacy persist, the path toward **data-driven precision oncology** is clear. Our proposed frameworks provide the necessary blueprint to transition from conceptual AI to real-world clinical impact, ultimately improving the quality of life for thyroid cancer patients worldwide.

## Figures and Tables

**Table 1:** Comparison of AI models for thyroid cancer applications.

Model Name	Authors (Citation No.)	Publication Year	Application Type	Dataset (Setting, n)	Accuracy/AUC	Strengths	Limitations
BETNET	[11]	2021	Diagnosis (US)	Single-center retrospective (n = 1250)	AUC 0.922 (0.79 for small nodules)	Real-time US; high specificity (92.2%); portable	Lower accuracy for subcentimeter nodules
TCS-CNN with AD-MIL	[18]	2021	Diagnosis (Cytopathology)	Multi-center WSI cohort (n = 3891)	97% accuracy (Bethesda)	Reduces indeterminate diagnoses by 40%; grad-CAM visual explanations	Requires manual filtering of non-diagnostic patches
ThyNet	[19]	2022	Diagnosis (Multimodal: US + Cytology)	Multi-center Prospective cohort (n = 4305)	AUC 0.922; 95% NPV	Reduces FNAs by 30%; outperforms radiologists	Preliminary; needs further validation
XAI-LIME	[20]	2024	Prognosis	Retrospective cohort with external validation (n = 850)	96% external validation accuracy	Explainable RF; identifies Tg & LNR; high clinical interpretability	High GPU demand; limited use in low-resource settings

**Table 2:** Comparison of proposed AI models for thyroid cancer management.

Model Name	Target Setting	Diagnostic Core	Prognostic Engine	Strategic Clinical Goal	Cost-Effectiveness	Limitations
ThyroSight-Prognos	Tertiary hospitals, research settings	TCS-CNN + Attention-based MIL (on FNAC/histology WSIs)	Random Forest using clinicopathological, biochemical, and genomic data	High-precision prognostic assessment	High upfront cost, but savings via optimized treatment; federated learning and cloud processing improve scalability	Hardware- intensive; complex workflow; suitable for advanced centers
SonoPredict-AI	Primary care, endocrinology clinics	BETNET (real-time ultrasound image analysis)	Random Forest using US and basic clinical data	Cost-effective screening & triage	Low-cost deployment; real-time assessment; reduces unnecessary FNAs	Limited depth in prognostication; reliance on US quality

**Table 3:** Projected computational budget and operational infrastructure requirements.

Computational Aspect	ThyroSight-Prognos (Tertiary/Specialized)	SonoPredict-AI (Primary/Mobile)
Core AI Paradigm	Foundation model (large-scale)	Foundation model (lightweight)
Target Hardware Spec	High-end multi-GPU cluster (e.g., 2× NVIDIA A100/H100)	Mid-range GPU (e.g., RTX 4090) or cloud-based CPU
Expected Latency	~5–10 min (comprehensive multimodal pipeline)	Sub-second (real-time US triage and simple risk score)
Model Architecture	Multimodal (US + WSI + genomics)	US-focused + basic EMR data
Interpretability (XAI)	Grad-CAM/multi-layer attention	LIME/SHAP feature importance
Infrastructure Mode	On-premise GPU cluster (H100)	Edge device/secure cloud
Feedback Mechanism	RLHF via expert clinicians	Automated health system cycle
Primary Clinical Value	Minimized recurrence & re-operation	Reduced unnecessary biopsies

## Data Availability

No new data were created in this study.

## Abbreviations

American Thyroid Association (ATA), Anaplastic Thyroid Carcinoma (ATC), area under the curve (AUC), artificial intelligence (AI), Artificial Neural Network (ANN), Attention-based Deep Multiple Instance Learning (AD-MIL), Body Mass Index (BMI), Central Lymph Node Metastasis (CLNM), Convolutional Neural Networks (CNNs), Decision Tree (DT), deep learning (DL), differentiated thyroid cancer (DTC), explainable AI (XAI), extranodal extension (ENE), extrathyroidal extension (ETE), eXtreme Gradient Boosting (XGBoost), fine-needle aspiration (FNA), fine-needle aspiration cytology (FNAC), Follicular Thyroid Carcinoma (FTC), Follicular Variant of Papillary Thyroid Carcinoma (FV-PTC), Gradient Boosting Decision Tree (GBDT), Gradient-weighted Class Activation Mapping (Grad-CAM), Inductive Logic Programming (ILP), K-Nearest Neighbors (KNN), Learning Health Systems (LHS), Local Interpretable Model-agnostic Explanations (LIME), Lymph Node Metastasis (LNM), lymph node metastasis ratio (LNR), lymph nodes (LNs), Lymphocyte-to-Monocyte Ratio (LMR), machine learning (ML), Medullary Thyroid Carcinoma (MTC), Multi-Layer Perceptron (MLP), negative predictive value (NPV), Neutrophil-to-Lymphocyte Ratio (NLR), Papillary Thyroid Carcinoma (PTC), Platelet-to-Lymphocyte Ratio (PLR), Probabilistic Neural Network (PNN), Prognostic Nutritional Index (PNI), Random Forest (RF), Reinforcement Learning from Human Feedback (RLHF), SHapley Additive exPlanations (SHAP), thyroglobulin (Tg), thyroglobulin antibodies (TgAb), Thyroid Imaging Reporting and Data System (TI-RADS), Thyroid Stimulating Hormone (TSH), Thyroid-Cytopathology-Specific CNN (TCS-CNN), ultrasonography (US), whole-slide images (WSIs).
